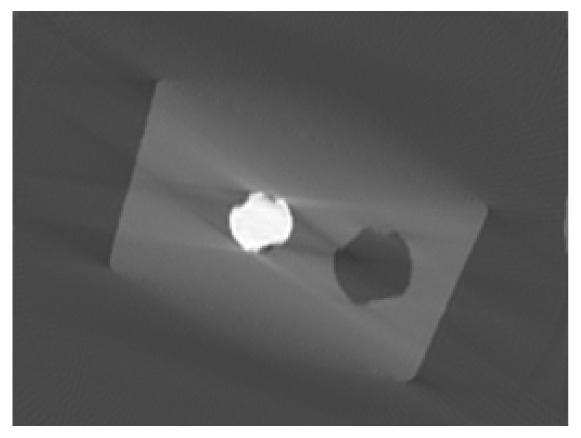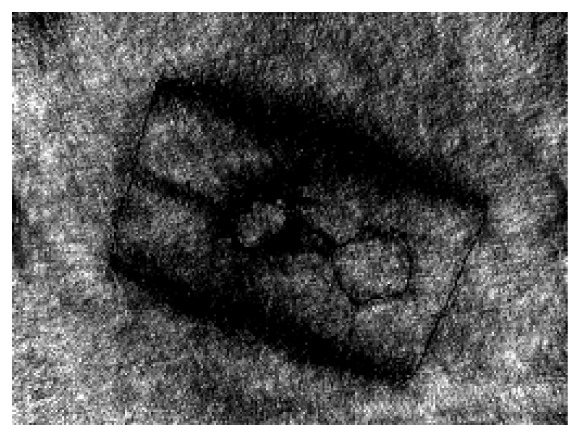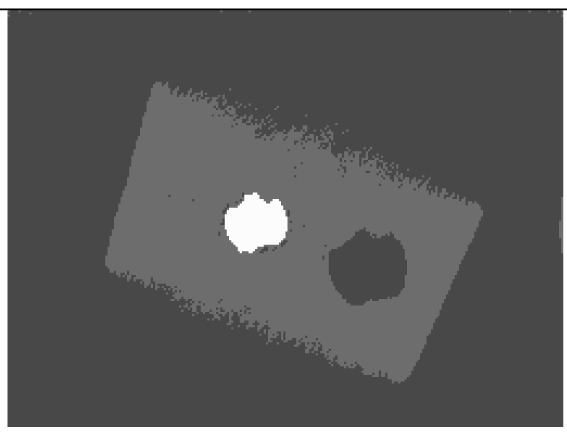# Probabilistic Evaluation of Three-Dimensional Reconstructions from X-Ray Images Spanning a Limited Angle

**DOI:** 10.3390/s130100137

**Published:** 2012-12-21

**Authors:** Anja Frost, Eike Renners, Michael Hötter, Jörn Ostermann

**Affiliations:** 1 Institut für Innovationstransfer, University of Applied Sciences and Arts, Ricklinger Stadtweg 120, Hannover, 30459, Germany; E-Mails: eike.renners@stud.fh-hannover.de (E.R.); michael.hoetter@fh-hannover.de (M.H.); 2 Institut für Informationsverarbeitung, Gottfried Wilhelm Leibniz Universität Hannover, Appelstraße 9A, Hannover, 30167, Germany; E-Mail: ostermann@tnt.uni-hannover.de

**Keywords:** X-ray, computed tomography, discrete tomography, three-dimensional image reconstruction, limited data, Dempster-Shafer theory, data fusion, probability calculus

## Abstract

An important part of computed tomography is the calculation of a three-dimensional reconstruction of an object from series of X-ray images. Unfortunately, some applications do not provide sufficient X-ray images. Then, the reconstructed objects no longer truly represent the original. Inside of the volumes, the accuracy seems to vary unpredictably. In this paper, we introduce a novel method to evaluate any reconstruction, voxel by voxel. The evaluation is based on a sophisticated probabilistic handling of the measured X-rays, as well as the inclusion of a priori knowledge about the materials that the object receiving the X-ray examination consists of. For each voxel, the proposed method outputs a numerical value that represents the probability of existence of a predefined material at the position of the voxel while doing X-ray. Such a probabilistic quality measure was lacking so far. In our experiment, false reconstructed areas get detected by their low probability. In exact reconstructed areas, a high probability predominates. Receiver Operating Characteristics not only confirm the reliability of our quality measure but also demonstrate that existing methods are less suitable for evaluating a reconstruction.

## 1. Introduction

First and foremost, computed tomography was introduced for clinical diagnostics. To an increasing degree, it is also used for quality assurance in the production and maintenance of man-made objects. As it generates a three-dimensional reconstruction of the inside of the object from large series of X-ray images, inner structures such as casting defects or cold soldered connections become visible. Moreover, exact measurements of the shape are feasible. But the very use in quality assurance demands reconstructions that are absolutely true to original. To calculate such reconstructions, it is necessary to provide many X-ray images from different angles of vision. Normally, the object is turned 360° while doing X-ray.

**Figure 1.**
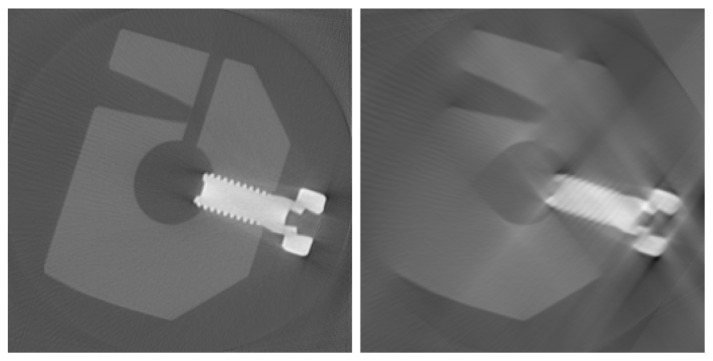
Cross sections through the reconstructed volumes from series of measured cone beam X-ray images spanning 360° **(left image)** and 100° **(right image).** They show an aluminium object with steel screw. The reconstructed density is proportional to the grey.

In some applications, it is not possible to turn the object 360°. For example, if the shape is bulky and stops the rotation in the computer tomography scanner, then the number of X-ray images is reduced. Mathematically speaking, the reconstruction problem is underdetermined. The reconstruction volume can only be estimated. For the most voxels, this estimation differs drastically from the original. The reconstructed object seems to be blurred (shown in Figure 1).

There are many reconstruction algorithms that try and rectify the reconstructed objects. Generally, the lack of X-rays is compensated by including additional information; a priori knowledge about the shape or about the materials that the object receiving X-ray consists of, *i.e.*, the specific density of each material. Most solutions that deal with knowledge about the shape fit a parametric model or an atlas to the reconstructed object. They get useful results, as shown for example in [1,2]. Others handle fragmentary knowledge about the shape demanding smoothness of the surface and combine knowledge about the materials [3,4]. It becomes more difficult to improve the reconstruction of any object if there is only knowledge about the materials available [5]. Our aim is to evaluate a given reconstructed volume, regardless of the algorithm that generated it.

Topical works express the reconstruction quality of a voxel by gradient based values [6–8] or Gibbs priors [9]. All these approaches exploit the densities of neighbouring voxels only. They are unsuited to detect bad areas in reconstructions from a limited angle range, since these areas are not remarkably sharp. Others evaluate a voxel by the difference between its reconstructed density and the next a priori known material density. In this way, test objects that consist of one solid material can be reconstructed [10,11]. When the test object is composed of several materials, the quality measure fails, because some voxels get a material density by mistake. For example, the test object in Figure 1 (on the right) is blurred. Voxels in the air near the steel screw wrongly yield the density of aluminium.

The evaluation proposed in this paper includes the measurement as well as a priori knowledge about the materials. For each voxel, the reconstructed density is replaced by each a priori known material density. For each replacement, it is reviewed how many X-rays support the current material. In so doing, we estimate a discrete probability distribution over material. Then, we apply Dempster's rule of combination, especially in the interpretation of Yager, to combine each probability value and get a new probability distribution, in which the reconstruction quality is reflected. In case the quality is poor, the distribution spreads out. The better the quality is, the more the distribution concentrates and the more its maximum increases. Finally, we pick out the maximum as quality measure, which we name “approbatio”. Since it is a probability, it provides a uniform basis to validate any reconstruction.

In Section 2 we give a brief introduction to the classical reconstruction techniques. Then, in Section 3 we enter into the question of how to include a priori knowledge. In Section 4 the evaluation is described. We present and discuss our experimental results in Section 5. A few remarks conclude the paper.

## 2. Reconstruction Technique

The grey value *y_p_* of pixel *p* in a measured X-ray image is equal to the line integral of the density *x* (*l*) along the ray path *l* (assuming a monochromatic X-ray source and ignoring scattering).

(1) $$y_{p}=\int x\;(l)\;\text{dl}$$

To calculate a reconstruction of the object examined by X-ray in a quantised grid, the Equation (1) changes into Equation (2). Here, *V* is the number of voxels in the whole volume, *x_υ_* represents the density of voxel *υ*, and *w_υp_* is a weighting factor that depends on the distance between the *p^th^* ray and the center of the *υ^th^* voxel. When the ray exactly hits the voxel, *w_υp_* equals one. When the distance increases, *w_υp_* drops. If there is no contribution of the ray to the voxel at all, *w_υp_* amounts to zero. The calculation of *w_υp_* is treated in [12].

(2) $$y_{p}=\sum_{\upsilon =1}^{V}w_{\upsilon p}x_{\upsilon }$$

There are several techniques to calculate the densities *x_υ_* from the measured pixel values *y_p_*, listed very comprehensively for example in [13]. These techniques can be divided into two categories: On the one hand, there are analytical methods, which involve all X-ray images to generate one reconstruction. On the other, there are iterative methods that also involve all X-ray images, but one after the other, *i.e.*, step by step. In each step, a correction for the current reconstruction is calculated. The correction equations of the most referred iterative reconstruction algorithms are based on minimisation of the cost function in Equation (3) [14].

(3) $$\text{cost}=\sum_{p}{\;(y_{p}-\sum_{\upsilon =1}^{V}w_{\upsilon p}x_{\upsilon })}^{2}$$

## 3. Including a Priori Knowledge

Some existing works, e.g., [3] and [15], extend the cost function in Equation (3) with an additive term that includes a priori knowledge. In so doing, they create their own reconstruction technique. For our aim to tackle the evaluation of any given reconstruction, we pursue a related strategy: For a selected voxel *s* the actual density *x_s_* is replaced successively with each predefined material density *m_d_* (with *d* = 1, …, *D* and *D* is the number of different materials). The rest of the volume is unchanged. Separately for each inserted material density *m_d_*, we calculate the projection error *e_sp_* (*m_d_*) of each X-ray *p* crossing exactly voxel *s*.

(4) $$e_{\text{sp}}\;(m_{d})=y_{p}-\sum_{\begin{array}{c} \upsilon =1\\ \upsilon \neq s \end{array}}^{V}(w_{\upsilon p}x_{\upsilon })-w_{sp}m_{d}$$

In the best case, the projection error *e_sp_* (*md*) equals zero, *i.e.*, for the current ray *p* the Equation (2) is fulfilled. In contrast, a high projection error indicates an imperfect reconstruction or a material that does not fit the measurement, or both.

## 4. Evaluation

### 4.1. Analysis of the Projection Error

In the following, we take the projection error as random variable *e_s_*. By consulting all X-rays that pass through a selected voxel *s*, we can record a histogram of projection error and estimate a continuous probability distribution *p* (*e_s_*), or more precisely one probability distribution per material density, *i.e.*, *p* (*e_s_* ∣ *m_d_*).

**Figure 2.**
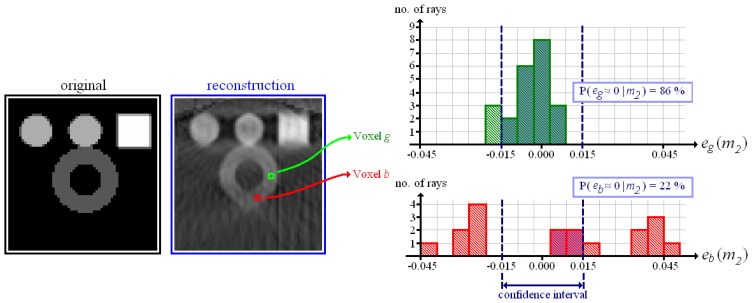
Test object and its reconstruction on the **(left).** Histograms of projection error on the **(right)** measured at the position of the “good” reconstructed voxel *g* and the “bad” reconstructed voxel *b*.

The test object shown in Figure 2 is made up of four different materials: *m*_1_ = 0.00 (representing air), *m*_2_ = 0.03 (hollow sphere), *m*_3_ = 0.06 (solid spheres) and *m*_4_ = 0.09 (cube). Exemplarily, we examine the histograms measured at the positions of two voxels that are both situated inside of the hollow sphere, and in both cases we inserted the very material density of the hollow sphere *m*_2_ = 0.03. Voxel *g* is evidently “good”. Here, most X-rays yield a projection error around zero. At the position of voxel *b* (as well as in its neighbourhood) the reconstruction is “bad”. The histogram of projection error spreads out. To sum up, the shape of the histogram gives an indication of the local reconstruction quality.

### 4.2. Extraction of a Probability Value

By counting the rays that hold a projection error in a defined confidence interval around zero, we estimate the probability *P* (−*δ* < *e_s_* < *δ* ∣ *m_d_*): a probability of an insignificant projection error under the condition that the material density *m_d_* exists at the position of voxel *s* while doing X-ray. We assume that an insignificant projection error is achieved if the voxel *s* cannot change to another material, neither *m_d_*_+1_ nor *m_d_*_−1_. *δ* results from the difference of material densities in Equation (5).

(5) $$\delta =\underset{1⩽d⩽D-1}{\min}\left(\frac{m_{d+1}-m_{d}}{2}\right)$$

In the following, we use the notation in Equation (6).

(6) $$P\;(-\delta <e_{s}<\delta |m_{d})=P_{s}\;(m_{d})$$

Note that

(7) $$\sum_{d=1}^{D}P_{s}(m_{d})\leq 1$$

is given, which leads to the use of the Dempster-Shafer theory (Section 4.4).

### 4.3. Discrete Distribution of Conditional Probabilities

**Figure 3.**
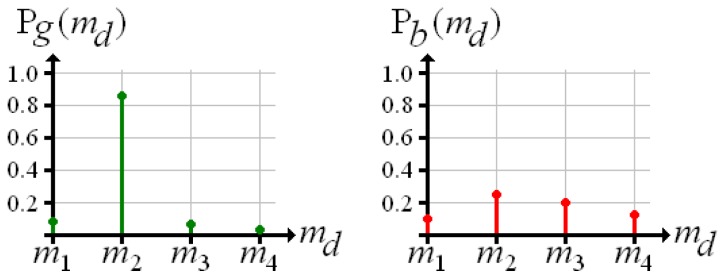
Discrete probability distributions over material density at the positions of voxel *g* and *b* according to Figure 2.

Now, for each voxel there is one probability value per material density. Exemplarily, Figure 3 shows the probability distributions over material density measured at the positions of voxel *g* and voxel *b* (according to Figure 2). For the “good” reconstructed voxel *g*, the material density of the hollow sphere achieves a high probability, whereas the probability of any other material is low. In the distribution of voxel *b* there is no distinct maximum.

### 4.4. Interpretation in the Sense of Dempster–Shafer

**Figure 4.**
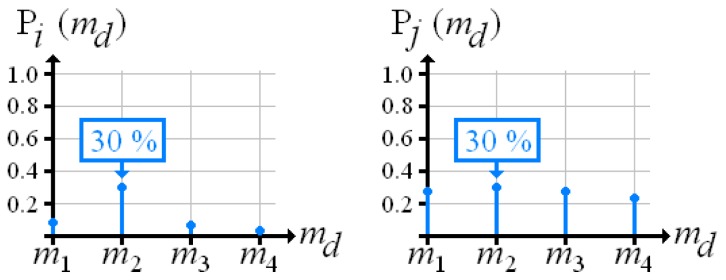
Probability distributions over material density at two voxels *i* and *j*.

The discrete probability distribution over material density already retains all the information required for determining quality Yet for practical applications, it is more advantageous to handle one numerical value that expresses the reconstruction quality The maximum of the distribution seems to be suitable, but it does not include conflicts, as demonstrated in Figure 4. Here, two different voxels *i* and *j* hold a maximum probability, which is 0.3 at material density *m*_2_. For voxel *i* this maximum stands out; the insertion of any other material produces extremely low probabilities. In contrast, for voxel *j* each material has approximately equal probabilities.

To keep conflicts, we apply the Dempster-Shafer theory [16,17]. Here, the basic idea is that a measured probability *P* (*A*) expresses how certain the event *A* occurs, but 1 − *P* (*A*) does not inform about the complementary event. Instead, 1 − *P* (*A*) is specified as degree of ignorance. In the same sense, our probability value *P_s_* (*m_d_*) was measured while inserting material density *m_d_*, and therefore provides information about *m_d_* only, not about other materials. Under the terms of Yager [17], each probability value *P_s_* (*m_d_*) of a voxel becomes fused with the degree of ignorance of each other material *m_c_* that the object examined by X-ray consists of Equation (8).

(8) $$P_{s,\text{fused}}\;(m_{d})=P_{s}\;(m_{d})\cdot \prod_{\begin{array}{c} c=1\\ c\neq d \end{array}}^{D}\left(1-P_{s}(m_{c})\right)$$

**Figure 5.**
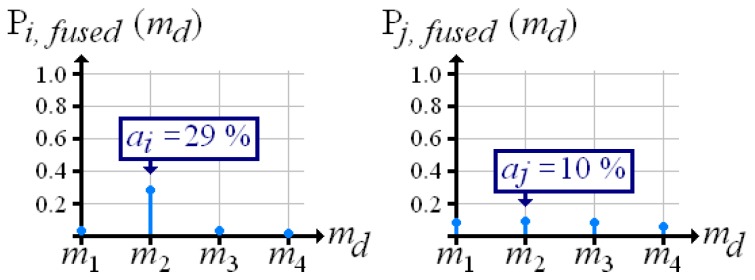
Fused probability distributions over material density according to Figure 4.

Figure 5 shows the fused probability distributions over material density that result from Figure 4. Now, the conflict-ridden voxel *j* holds a lower maximum than voxel *i*.

### 4.5. Proposed Quality Measures

We introduce the maximum of the fused probability distribution *P_s,fused_* (*m_d_*) as quality measure, which we name approbatio *a_s_* in Equation (9).

(9) $$a_{s}=\underset{1⩽d⩽D}{\max}P_{s,\text{fused}}\;(m_{d})$$

In addition to the evaluation by the voxel, we use the average approbatio *a* in Equation (10) to express the reconstruction quality of a whole volume.

(10) $$a=\frac{1}{V}\sum_{\upsilon =1}^{V}a_{\upsilon }$$

## 5. Results and Discussion

### 5.1. Test Environment

**Figure 6.**
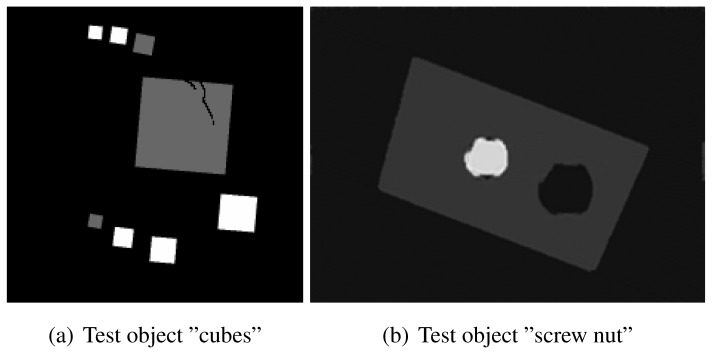
Cross sections through both test objects perpendicular to the rotation axis.

The experiment deals with two different test objects, both situated in cone beam geometry. The first test object consists of eight cubes in which one cube is cracked (pictured in Figure 6(a)). We briefly name it “cubes”. From this test object, 200 X-ray images spanning 360° are available. Each image has the size of 200 × 40 pixels. The reconstructed volume contains 200 × 200 × 40 voxels. We executed the iterative reconstruction algorithm SART [12] up to the 12*th* iteration step. After each iteration step, we evaluated the whole volume providing the three materials of which the test object consists: *m*_1_ = 0.0, *m*_2_ = 0.2, *m*_3_ = 0.4.

The second test object, as displayed in Figure 6(b), is described as “screw nut”. It is made of aluminium (*m*_2_ = 0.0035) and contains a steel screw (*m*_3_ = 0.015). We generated three different series of X-ray images spanning a limited angle; 90° (64 images), 130° (92 images) and 150° (107 images). The standard deviation of the grey value *y_p_* is about 0.001, which is caused by noise. Each image has a resolution of 256 × 256 pixels. From each series, we reconstructed the test object using SART and evaluated the reconstructed volume after the 12*th* iteration step. The volume contains 192 × 256 × 256 voxels.

### 5.2. Visual Analysis of Approbatio

The first column in Table 1 shows cross sections through the reconstructed volume of the test object “cubes” after 3, 6 and 12 iteration steps. As can be seen, the reconstructions are close to the original and their sharpness is increasing with the iteration number. The corresponding approbatio, as pictured in the second column, is also increasing with iteration number. Generally at the surface the reconstruction is difficult, because some rays prefer the material of the cube while others favour air, which then results in a low approbatio. Depending on the amount of material and air, the approbatio value varies, which is reflected in dashed lines along the surface. Beyond the surface, approbatio is consistently high. The most likely material density *m_d_* (*a_s_*) (*i.e.*, the material that belongs to the approbatio *a_s_* (*m_d_*)) is shown in the third column in Table 1. Obviously, it is close to the original in Figure 6(a).

Similarly, Table 2 shows the results of the test object “screw nut” after 12 iteration steps. The fewer X-ray images are available, the more the reconstructed test object is blurred and affected by artefacts. In case of 130° as well as 150°, the bad reconstructed areas get identified by their low approbatio. When the X-ray images span only 90°, the reconstructed test object differs drastically from the original in Figure 6(b). Hence, hardly any voxel yields a histogram of projection error that is concentrated. Even for the few good reconstructed voxels, the histograms spread out because the surrounding is extremely affected by artefacts. At this point, the quality measure fails, but it fails to the right side: The corresponding approbatio is low.

### 5.3. Average Approbatio

**Figure 7.**
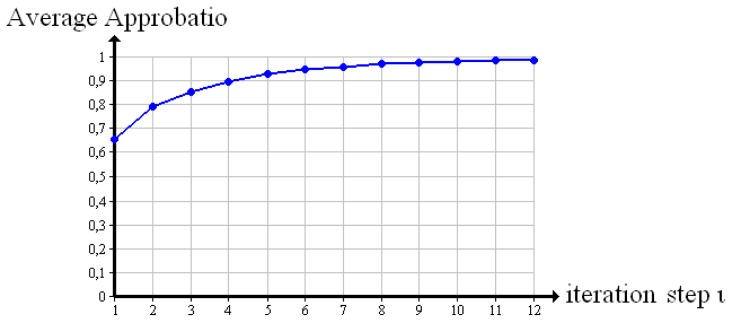
Average approbatio course while reconstructing the test object “cubes” in 360°.

The apparent increase in quality while reconstructing the “cubes” is confirmed by the average approbatio *a*.Figure 7 shows the course the average approbatio takes from iteration step *ι* = 1 to iteration step *ι* = 12. It goes up to 97%. The original verifiable reaches 100%.

The expansion of bad reconstructed areas that was already observed in Table 2 find expression in the average approbatio, which is only 35.1% (90°), 56.1% (130°) and 58.3% (150°).

### 5.4. Convergence

In the following, we analyse the behaviour of our evaluation method when the number of iteration steps approaches infinity. For each voxel *s* the original value *o_s_* is compared with the most likely material density *m_d_* (*a_s_*). Introducing a sample space *K* = {*k*_0_, *k*_1_}, in which *k*_1_ represents a correct material assignment and *k*_0_ a mistake, it follows:

(11) $$\begin{array}{ccc} k_{s}=k_{0} & \text{if} & m_{d}\;(a_{s})\neq o_{s} \end{array}$$

(12) $$\begin{array}{ccc} k_{s}=k_{1} & \text{if} & m_{d}\;(a_{s})=o_{s} \end{array}$$

Then, we define an ideal quality measure demanding *k*_0_ = 0 and *k*_1_ = 1, and observe the quadratic mean *E* [(*a_s_* − *k_s_*)^2^] of all voxels in the reconstruction of the “cubes” from iteration step *ι* = 1 to iteration step *ι* = 12. By this we analyse the convergence of our evaluation method in Equation (13).

(13) $$\underset{\iota \to \infty }{\lim}E\;[{\left(a_{s,\iota }-k_{s,\iota }\right)}^{2}]=0$$

**Figure 8.**
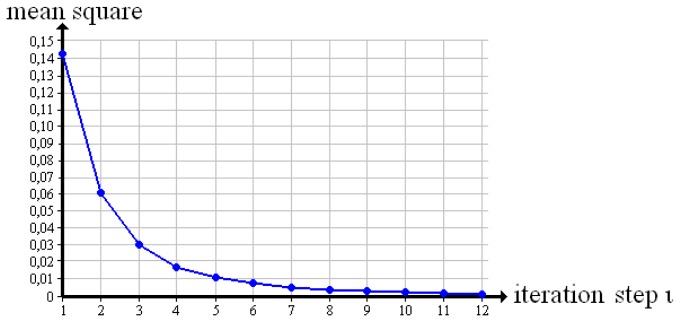
Convergence test.

Figure 8 shows the result for the “cubes”. The quadratic mean tends towards zero. In the present case, our evaluation method converges.

### 5.5. Receiver Operating Characteristics of Different Quality Measures

**Figure 9.**
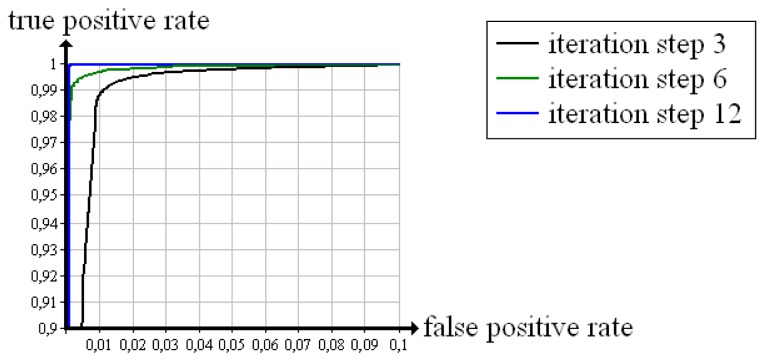
Receiver Operating Characteristics of approbatio evaluating the reconstruction of the test object “cubes”.

We define the Neyman-Pearson criterion of *N* = {*n*_0_, *n*_1_}

(14) $$\begin{array}{ccc} n_{s}=n_{0} & \text{if} & a_{s}<\lambda \end{array}$$

(15) $$\begin{array}{ccc} n_{s}=n_{1} & \text{if} & a_{s}\geq \lambda \end{array}$$

to binarise approbatio *a_s_* on the basis of a varied threshold value λ (and 0 ⩽) λ ⩽ 1;. The true positive rate *P* (*n*_1_ ∣ *k*_1_) results from counting all voxels that fulfil the conditions in Equations (12) and (15). Similarly, the false positive rate *P* (*n*_1_ ∣ *k*_0_) results from counting all voxels that fulfil the conditions in Equations (11) and (15). Using these rates, we recorded the Receiver Operating Characteristics of the “cubes” in Figure 9. From iteration step 3 to iteration step 12, the curve moves to a higher true positive rate. The evaluation becomes more and more reliable. In the 12*th* iteration step, the curve is nearly ideal.

The 12*th* iteration step is also tested with two existing evaluation methods; Censor [10] uses a quality measure that is based on the difference between the reconstructed density of a voxel and the next a priori known material density. In the following, we briefly call it “difference”. Batenburg and Sijbers [8] discretise the reconstructed volume via thresholding and subsequently define all voxels whose gradient comes to zero as “good”, the others as “bad”. We name this determination technique “binarised gradient”. Furthermore, we tested the 12*th* iteration step with our own evaluation method skipping the fusion (“approbatio without fusion”).

**Figure 10.**
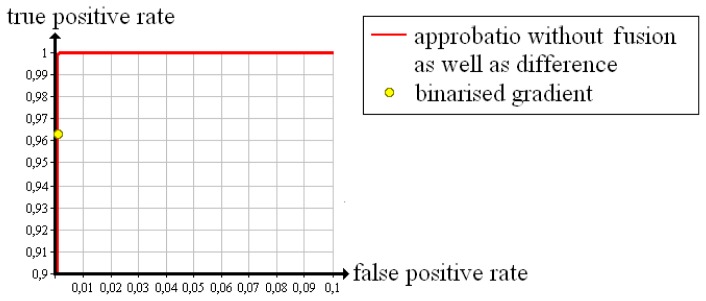
Receiver Operating Characteristics of different quality measures evaluating the reconstruction of the test object “cubes” after 12 iteration steps.

In Figure 10, the Receiver Operating Characteristic that follows from approbatio without fusion is pictured in red. The evaluation via difference yields an identical curve, *i.e.*, both quality measures are suited to evaluate the reconstructed test object. The yellow dot results from determining with binarised gradient. The false positive rate equals zero. The true positive rate amounts to 96.3%. Compared with the other quality measures, the binarised gradient is marginally worse, but for safety reasons a low false positive rate is more important than a high true positive rate. Hence, each quality measure reliably evaluates the test object.

**Figure 11.**
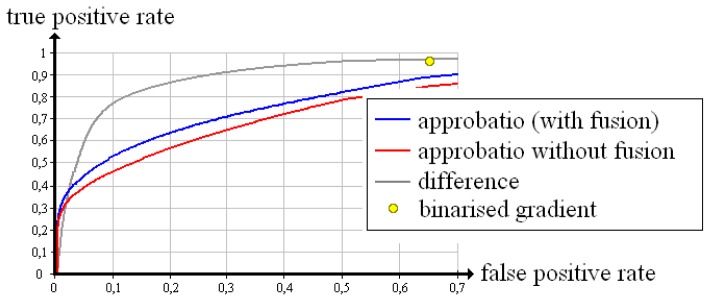
Receiver Operating Characteristics of different quality measures evaluating the reconstruction of the test object “screw nut” from X-ray images spanning 90°.

**Figure 12.**
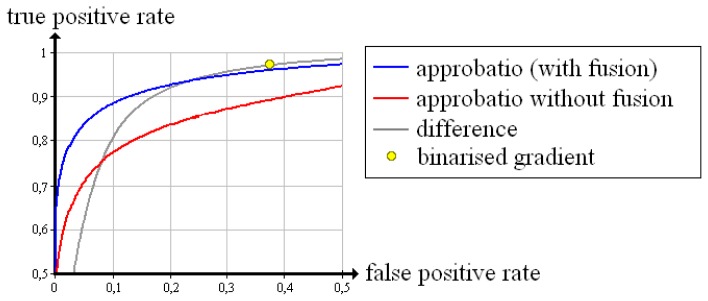
Receiver Operating Characteristics of different quality measures evaluating the reconstruction of the test object “screw nut” from X-ray images spanning 130°.

**Figure 13.**
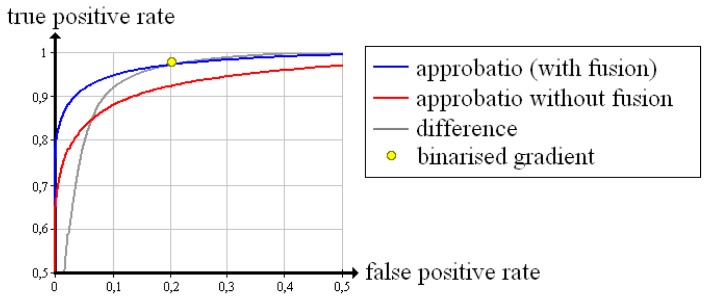
Receiver Operating Characteristics of different quality measures evaluating the reconstruction of the test object “screw nut” from X-ray images spanning 150°.

Equally, Figures 11, 12 and 13 summarise the Receiver Operating Characteristics measured with the “screw nut”. It becomes visible that the true positive rate of each quality measure is reduced by the limitation of the angle range and noise. Yet the difference turns out to be less suitable for evaluation: There is no threshold λ that reaches a false positive rate of zero (apart from the maximum threshold, which also produces a true positive rate of zero). The binarised gradient outputs a high false positive rate that is 65.3% in case of 90°, 38.0% for 130° and 19.8% for 150°. Approbatio without fusion keeps a false positive rate of zero while the true positive rate amounts to 22.9% (90°), 52.5% (130°) and 66.2% (150°). The fusion actually improves the evaluation. The true positive rate increases up to 26.2% (90°), 66.1%(130°) and 79.7% (150°).

## 6. Conclusions

In this paper, we have shown a sophisticated analysis of X-ray images. By handling each X-ray crossing a selected voxel separately, very small inconsistencies between the reconstruction and the measurement get revealed. Considering all these X-rays in equal weighting, we record a histogram. This histogram reflects the reconstruction quality. For an inaccurate reconstructed voxel, the histogram spreads out. For a generally good reconstructed voxel, the histogram is more concentrated around zero. By counting all rays in a defined confidence interval around zero, we get a probability value that expresses the current reconstruction quality.

We replace the reconstructed density of a selected voxel successively by the density of each a priori known material that the object examined by X-ray consists of. For each replacement the histogram is recorded. Hence, we are able to assign one probability value to each material. The resulting discrete probability distribution over material density expresses the reconstruction quality again. In good reconstructed areas, exactly the material density that really existed at the position of the selected voxel while doing X-ray stands out with a high probability. In a more inaccurate reconstructed area, there is no distinct maximum. We fuse the distribution under the terms of Yager, and choose the most likely material. The corresponding probability value is introduced as quality measure, called “approbatio”.

Our experiment deals with two different test objects that are typical for industrial applications. They are composed of several materials (e.g., aluminium and steel, surrounded by air) and analysed in cone beam geometry. We assume a monochromatic X-ray source and ignore scattering. The influence of noise is not analysed in detail.

For the first test object, ideal X-ray images of a 360° turn are available. From these images, we reconstructed the test object using SART and evaluated the volume voxel by voxel after 3, 6 and 12 iteration steps. When the reconstruction is true to the original, our evaluation method outputs high approbatio values. At the surface, the reconstruction is difficult, even after 12 iteration steps. Some rays prefer air, others favour the material of the object. These inconsistencies cause low approbatio values.

The main focus of our work is the problem of limited angle. We reconstructed a second test object from three different series of noisy X-ray images, each spanning a limited angle: 90°, 130° and 150°. The smaller the angle range is, the more the reconstructed test object is blurred. Our experimental results show that blurred areas reliably get detected by low approbatio values. For 130° and 150°, good reconstructed areas yield high approbatio values. When the X-ray images span only 90°, the reconstructed volume is highly corrupted. Almost in the whole volume the evaluation method outputs low approbatio values. This is a right-side failure.

For a further check of the reliability, we have recorded the Receiver Operating Characteristic of different quality measures; approbatio (with fusion), approbatio without fusion, difference and binarised gradient. For good reconstructions from ideal X-ray images, each approbatio (with fusion), approbatio without fusion and difference exactly achieves a true positive rate of 100% and a false positive rate of 0%. With the binarised gradient, the true positive rate amounts to 96.3%. By evaluating test objects that are examined by X-ray in a limited angle range, the advantage of approbatio becomes evident. Neither the difference nor the binarised gradient reaches a false positive rate of zero. By using approbatio without fusion, a false positive rate of zero is maintained while the true positive rate is 22.9% for the 90° case, 52.5% for 130°, 66.3% for 150°. Moreover, the probabilistic background of our evaluation method makes possible to use the Dempster–Shafer theory and detect conflicts. Then, the associated fusion improves the reliability of our evaluation method. The true positive rate increases up to 26.2% (90°), 66.1% (130°) and 79.7% (150°). At the same time the false positive rate still amounts to zero.

Furthermore, the average approbatio expresses the reconstruction quality of a whole volume. While reconstructing a test object, the average approbatio steadily increases towards a maximum quality that is 100% for ideal test objects examined by X-ray. Similarly, we observed the mean square over iteration steps, with the result that it tends to zero. The convergence of our evaluation method is confirmed.

In future works, we want to analyse the influence of the noise level in X-ray images in more detail.

## Figures and Tables

**Table 1.** Cross sections through the reconstructed volumes of the test object “cubes” and the corresponding evaluation. A higher value is always represented by a lighter greyness. The reconstructions as well as the most likely materials are displayed in the grey level window [0, 0.4], approbatio in [0.5, 1.0].

Iteration	Reconstruction	Approbatio *a_s_* (*m_d_*)	Most likely material
step 3	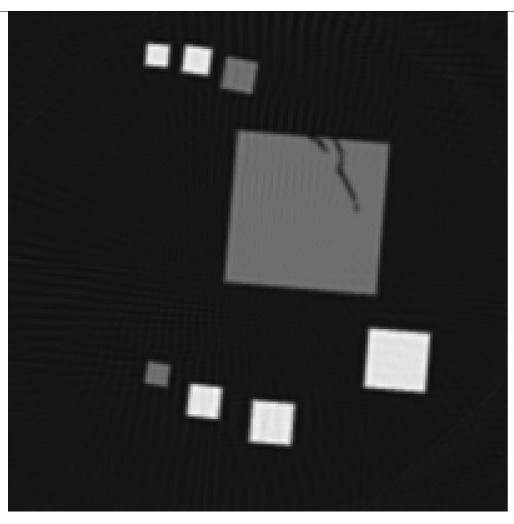	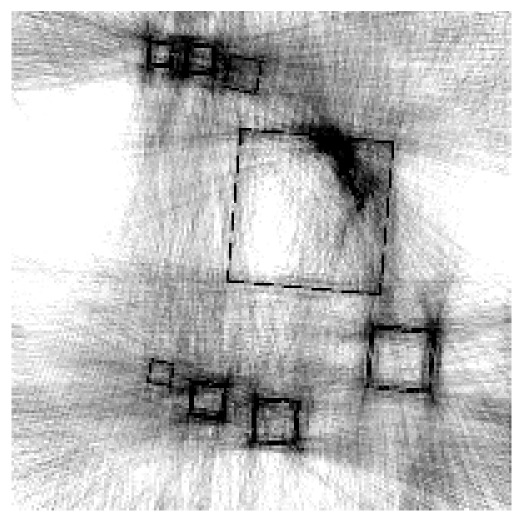	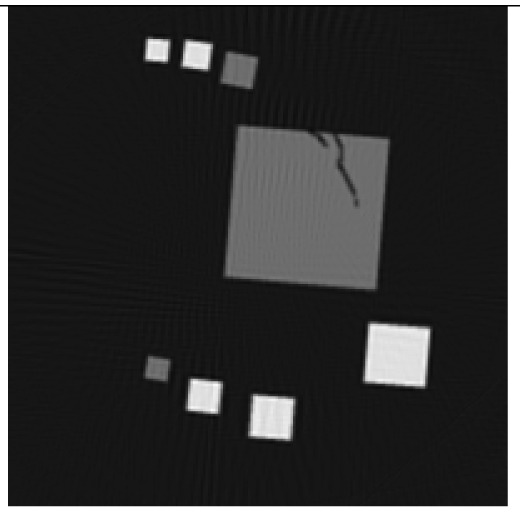
step 6	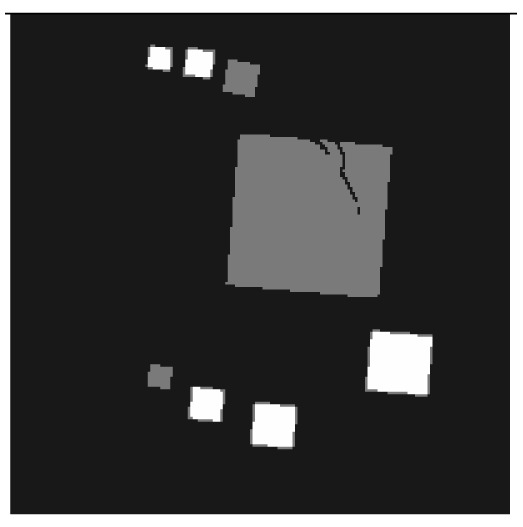	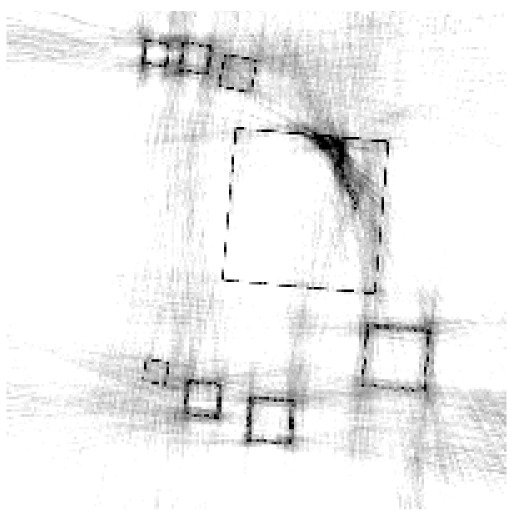	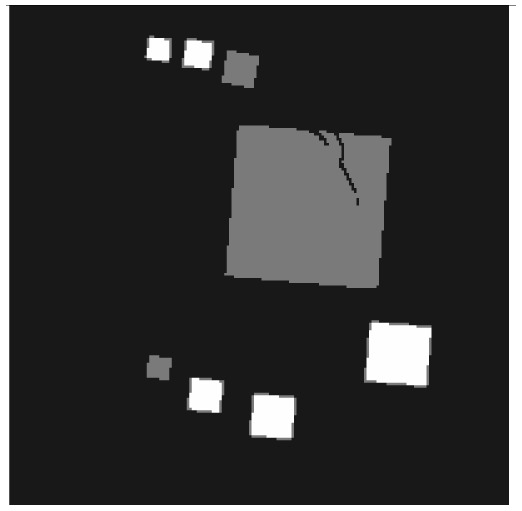
step 12	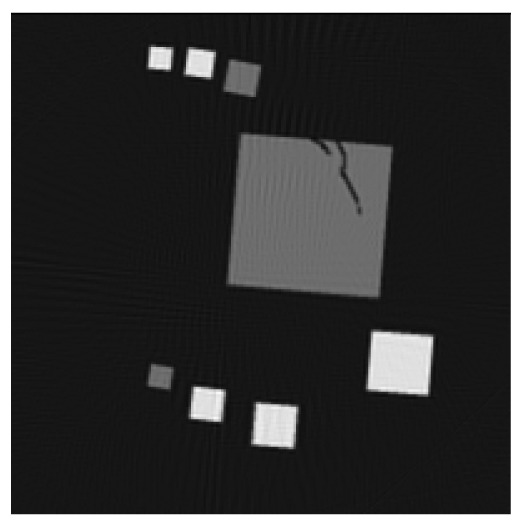	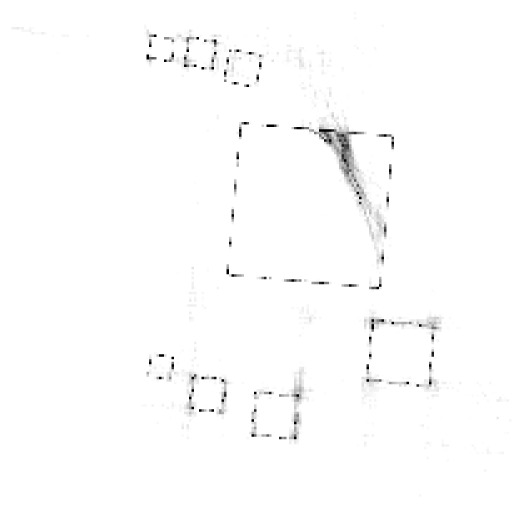	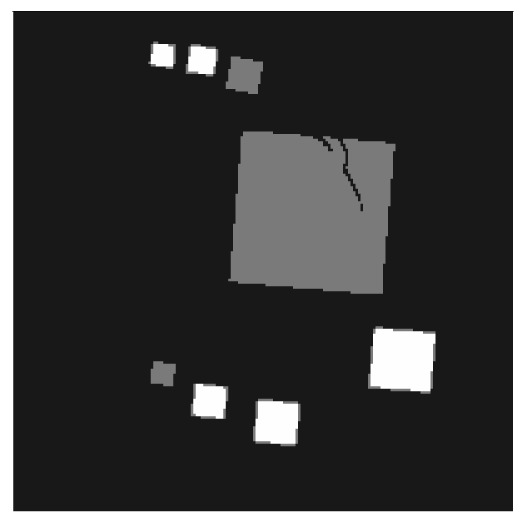

**Table 2.** Cross sections through the reconstructed volumes of the test object “screw nut” and the corresponding evaluation. A higher value is always represented by a lighter greyness. The reconstructions as well as the most likely materials are displayed in the grey level window [0, 0.015], approbatio in [0.5, 1.0].

Angle	Reconstruction	Approbatio *a_s_* (*m_d_*)	Most likely material
90°	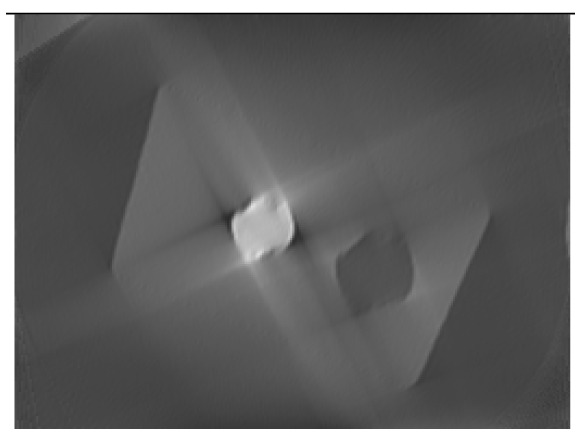	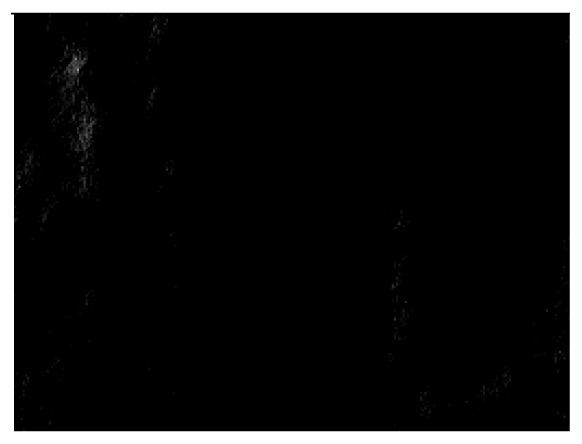	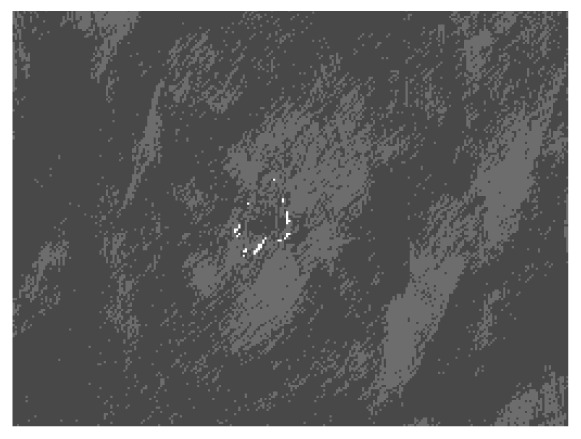
130°	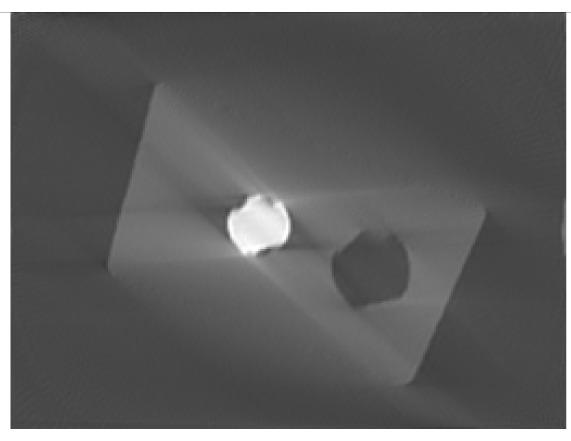	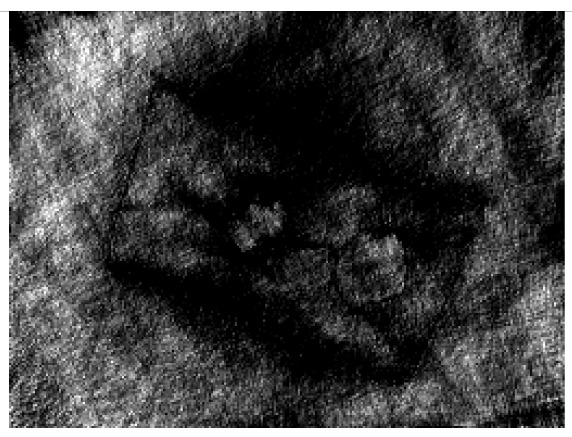	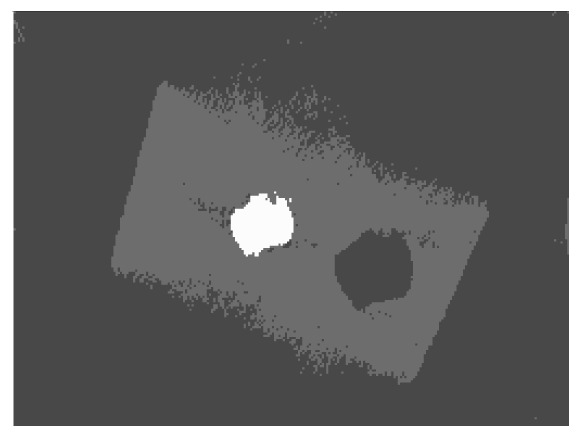
150°